# Biosynthesis, characterization, and antifungal activity of plant-mediated silver nanoparticles using *Cnidium monnieri* fruit extract

**DOI:** 10.3389/fmicb.2023.1291030

**Published:** 2023-11-20

**Authors:** Mingqi Ye, Wenwen Yang, Minxin Zhang, Huili Huang, Aiwen Huang, Bin Qiu

**Affiliations:** ^1^Fujian University of Traditional Chinese Medicine Fuzong Teaching Hospital (900TH Hospital), Fuzhou, China; ^2^Department of Clinical Pharmacy, 900TH Hospital of Joint Logistics Support Force of PLA, Fuzhou, China; ^3^College of Chemistry, Fuzhou University, Fuzhou, China

**Keywords:** AgNPs, *Cnidium monnieri*, green synthesis, characterization, antifungal

## Abstract

The present study describes a novel method for green synthesis of silver nanoparticles using *Cnidium monnieri* (CM-AgNPs). *Cnidium monnieri* fruit is an excellent anti tinea drug that can be used externally to treat superficial fungal infections in the human body. The aqueous ethanolic extract of *Cnidium monnieri* fruit was prepared and employed in the synthesis of stable silver nanoparticles via biological reduction method. The synthesis conditions of CM-AgNPs was systematically optimized using Box–Behnken design. CM-AgNPs were well characterized by UV-spectroscopy and X-ray powder diffraction (XRD), and it was confirmed that the synthesized particles were AgNPs. The possible functional groups required for the reduction and stabilization of CM-AgNPs in the extract were identified through FTIR spectrum. The size of CM-AgNPs structure was confirmed to be approximately 44.6 nm in polydisperse spherical shape through scanning electron microscopy (SEM), transmission electron microscopy (TEM), and laser dynamic light scattering (DLS). Further, the minimum inhibitory concentration 90% (MIC_90_) ratios values of Cm-AgNPs against *Trichophyton rubrum* (7 d), *T. mentagrophytes* (7 d) and *Candida albicans* (24 h) were 3.125, 3.125, and 0.78125 μg/mL, respectively, determined by the broth micro dilution method. Finally, the result was concluded that the synthesized AgNPs could be further evaluated in large scale as a potential human topical antifungal agent.

## 1 Introduction

In recent years, nanoparticles (NPs) have been widely used in biological, pharmaceutical, electronic, chemical and energy industries (Yoon et al., 2020; Jin Nam et al., 2021; Mobini et al., 2021; Targhi et al., 2021; Zhao et al., 2021; Wei et al., 2022). Due to the need to reduce or eliminate the use or generation of toxic and harmful substances in compounds, reduce pollution to the environment and develop more sustainable methods, biosynthesis of nanoparticles (NPs) using natural product extracts has been proposed as a harmless, rapid and efficient alternative synthesis route. As a safe and non-toxic method, biosynthetic silver nanoparticles (AgNPs) was used to produce biocompatible nanoparticles by using bioactive molecules with functions of reduction, capping and stabilization, which were suitable for many medical applications, such as antibacterial, anticancer, antioxidant, anti-diabetic, antiviral, and anti-inflammatory (Chinnasamy et al., 2019; Mehmood et al., 2020; Tian et al., 2020; Jang et al., 2022). Different parts of plants could be used to synthesize silver nanoparticles (Ma et al., 2021; Moorthy et al., 2021; Mirzaie et al., 2022; Abdel-Aty et al., 2023). Using plant extracts biosynthetic silver nanoparticles, AgNPs were covered with active ingredients from plants, which can not only stabilize AgNPs, but also enhance their antifungal activity (Robles-Martinez et al., 2019). Biosynthesized AgNPs can treat infections caused by *Trichophyton rubrum* in animals’ models, and after 14 days of treatment, the structure of the animal’s epidermis and dermis can be restored well, and the synthesized AgNPs have no risk of antimicrobial resistance or systemic side effects of other drugs (Abdallah et al., 2023).

Superficial infections, including cutaneous and mucocutaneous infections, are a common public health problem and can be caused by dermatophytes, candida, and Malassezia (Noites et al., 2023). Superficial fungal infections usually cause chronic and non-inflammatory lesions, long treatment course, easy relapse, pain, unattractive and other problems affecting the quality of life of patients (Abd-Elsalam and Abouelatta, 2023). A number of comorbidities such as diabetes, cancer, immune deficiency or peripheral artery disease may increase susceptibility to superficial fungi. Filamentous fungi *T. rubrum* and *T. mentagrophytes* were one of the most important pathogens causing dermatomycosis in the UK, Poland and Sweden, accounting for about 90% of cases (Nenoff et al., 2014). Although *Candida albicans* is a common fungal symbiont in the human microbiota, it may cause superficial skin, nails, and mucous membrane infections (Glazier, 2022). Long-term use of azoles, allylamines, and terbinafine in the treatment of dermatophytes may develop drug resistance and toxicity, leading to treatment failure (Sardana et al., 2023). The need to slow down and prevent the development of drug resistance and reduce side effects has prompted researchers to develop new alternative treatment drugs for superficial infections.

*Cnidium monnieri* (L.) Cuss, an annual plant in the umbelliferous family, is widely used as a traditional herb in China, Japan and Vietnam to treat various diseases. It has a wide range of pharmacological activities, the main component being osthole, which has an allergy-inhibiting (antipruritic) effect on the skin, as well as bacterial, fungal and viral inhibition (Munir et al., 2022). According to modern research, the mechanism of treating *T. rubrum* is that the water extract from *C. monnieri* can destroy the mycelial morphology and internal structure of *T. rubrum* and inhibit its growth (Yanyun et al., 2021). The extract of *C. monnieri* fruit contains various reducing components, including flavonoids, coumarins, phenolic acids, and lignans, which contribute to the reduction of silver nitrate (Wu et al., 2019; Ni et al., 2020). The complex components contained in the extract of *C. monnieri* fruit are coated on silver nanoparticles to prevent aggregation and facilitate the stability of silver nanoparticles. *C. monnieri* are widely distributed and have low prices, making it low-cost to synthesize AgNPs using them.

In this report, we innovatively prepared AgNPs using the extract of dried and mature fruit of the anti ringworm drug *C. monnieri*, using a simple biosynthesis method, and studied their activity against *T. rubrum*, *T. mentagrophytes*, and *C. albicans*. Compared with physical and chemical synthesis methods, the process of green synthesis of AgNPs using *C. monnieri* fruit extract is simple, does not use toxic reagents, and the process of preparation is relatively safe and environmental friendly. The synthesized CM-AgNPs are expected to be used in various dosage forms for superficial fungal infections in humans and animals, but further research is to be demonstated their efficacy.

## 2 Materials and methods

### 2.1 Materials

Silver nitrate purchased from Yida Technology (Quanzhou) Co. Ltd., concentration 0.1001 mol/L, diluted for use in the experiment. *C. monnieri* fruit was provided by the pharmacy of the No.900 Hospital of Joint Logistics Support Force of PLA. Deionized water was used to synthesize, extract and purify the nanoparticles. All chemicals used were analytical grade. *T. rubrum* (BNCC340195) and *T. mentagrophytes* (BNCC340405) were purchased as standard strains from Beijing BeNa Culture Collection. *C. albicans* was a clinically isolated strain, which was provided by the laboratory department of the No.900 Hospital of Joint Logistics Support Force of PLA. RPMI 1640 culture medium was purchased from Wuhan Boster Biological Technology Co. Ltd. Potato dextrose agar (PDA) was purchased from Wenzhou Kangtai Biotechnology Co. Ltd. Sabouraud dextrose agar (SDA) was purchased from Zhengzhou Antu Bioengineering Co. Ltd. Fluconazole and terbinafine were purchased from China National Institutes for Food and Drug Control.

### 2.2 Preparation of plant extracts

50 g of *C. monnieri* fruit was weighed and soaked in 500 mL of 75% ethanol for 0.5 h, then heated and refluxed extraction twice for 2 h each time. The extracted solution was combined and concentrated under reduced pressure until there was no alcohol taste. Pure water was added to dilute to 250 mL and filtered by 0.22 μm microporous membrane to obtain *C. monnieri* fruit extract, which was stored at −40°C.

### 2.3 Synthesis of CM-AgNPs

CM-AgNPs were synthesized with different concentrations of *C. monnieri* fruit extract (65, 132.5, 200 mg/mL). NaOH solution (0.1 M) was used to adjust the pH of *C. monnieri* fruit extract (8, 10, 12). 6 mL of pH-adjusted *C. monnieri* fruit extract was slowly dripped into 20 mL of silver nitrate at different concentrations (5, 12.5, 20 mM), and stirred continuously for 30 min at 700 rpm with the help of magnetic stirrers (Srećković et al., 2023). The resulting CM-AgNPs solution was centrifuged at 13,000 rpm for 20 min at 20°C. The supernatant was removed and the precipitate was redispersed in deionized water and centrifuged again at 13,000 rpm for 20 min. This process was repeated three times. Finally, the centrifugal precipitate was freeze-dried, weighed and redissolved with deionized water.

### 2.4 Systematic optimization of synthesis of CM-AgNPs

System optimization of CM-AgNPs was performed using a Box–Behnken design (BBD) with the help of Design Expert® ver. 13.0 software (Stat-Ease Inc., Minneapolis, USA). The three most influential factors, AgNO_3_ concentration, *C. monnieri* fruit extract concentration and pH value, were used as independent variables and tested at three different levels, as shown in Table 1. A total of 17 tests were recommended for the selected design. The particle size (nm) and polydispersity index (PDI) of the synthesized silver nanoparticles (CM-AgNPs) were analyzed in response. After putting the data into BBD, mathematical modeling was carried out to analyze the results. The optimum conditions for the synthesis of CM-AgNPs were determined by means of numerical desirable function and graphic optimization techniques.

**Table 1:** Response surface experiment design and results.

Runs	Concentration of AgNO_3_ (mM)	Concentration of *Cnidium monnieri* fruit extraxt (mg/mL)	pH	Particle size (nm)	PDI
1	12.5	132.5	10	74.87	0.2857
2	5	132.5	8	113.23	0.2412
3	12.5	200	12	83.18	0.2576
4	12.5	132.5	10	80.98	0.2916
5	12.5	132.5	10	83.36	0.3019
6	12.5	200	8	97.05	0.2734
7	20	65	10	139.43	0.4307
8	12.5	65	12	73.06	0.3765
9	5	200	10	130.7	0.2725
10	12.5	132.5	10	81.09	0.2816
11	20	132.5	12	87.84	0.2848
12	20	200	10	82.43	0.2978
13	12.5	132.5	10	89.27	0.3647
14	20	132.5	8	112.67	0.4023
15	12.5	65	8	103.33	0.2918
16	5	65	10	88.71	0.3443
17	5	132.5	12	129.9	0.2191

### 2.5 Chemical synthesis of bare AgNPs

Reference The preparation method of bare AgNPs and make appropriate modifications (Bharti et al., 2021; Fu et al., 2021; Jassim et al., 2022; Velgosova et al., 2022). 9.45 mg of NaBH_4_ Was weighed and added To 25 mL of NaOH solution (0. L Mol/L) To prepare a reducing agent. The 20 mL of AgNO_3_ solution and sodium citrate solution (As dispersant) with a concentration of 0.01 Mol/L each were added into The conical flask and stirred evenly, then 1 mL of The reducing agent solution Was added drop By drop, and The solution turned brownish black at room temperature for 5 Min. The resulting bare AgNPs solution Was centrifuged at 13,000 rpm for 20 Min at 20°C, and The precipitate Was rinsed three times with deionized water

### 2.6 Characterization of AgNPs

CM-AgNPs synthesized under optimal conditions and chemically synthesized bare AgNPs were scanned using a microplate reader (Infinite E Plex, Tecan Austria Gmbh, Austria) in the wavelength range of 200–800 nm. Fourier transform infrared (FTIR, Nicolet 6,700, Thermo Fisher, United States) spectra of CM-AgNPs and *C. monnieri* fruit extract were recorded using KBr particles. X-ray diffractometer (XRD, D8 Advance, Bruker, Germany) was used to analyze CM-AgNPs under diffraction conditions of copper target, Cu Kα radiation, measuring angle 2 θ = 5–90 °. SEM was used to observe the morphology of Cm-AgNPs and energy dispersive X-ray spectroscopy (EDX) results were obtained (Mira Lms, Tescan, Czech Republic). The shape and dimensions of CM-AgNPs were determined at 100 kV by TEM (Tecnai, Thermo Scientific FEI, United States). Particle size, PDI and zeta potential values of CM-AgNPs and bare AgNPs were recorded by dynamic light scattering (DLS) using a laser particle size analyzer (ZSU3100, Malvern Panalytical, Worcestershire, UK).

### 2.7 Antifungal assays

#### 2.7.1 MIC determination of *Trichophyton rubrum* and *Trichophyton Mentagrophytes*

The strains of *T. rubrum* and *T. mentagrophytes* were inoculated on PDA medium and cultured at 28°C for 7–14 days. The number of spores was determined by cell counting plate (177-112C, Watson, Japan), and the concentration was adjusted to 7.1 × 10^4^–1.1 × 10^5^ CFU/mL by RPMI 1640 medium dilution. MIC was determined according to CLSI-M38 document (Alexander et al., 2017a), RPMI 1640 culture medium was added to the 96-well plates and the sample was diluted to different concentrations using broth microdilution method (terbinafine was dissolved with DMSO, other drugs were dissolved with deionized water, DMSO content was 0.5%). In columns 1–9 of the 96-well plates, concentration of bare AgNPs lyophilized powder in nutritional broth was 250–0.98 μg/mL, concentration of *C. monnieri* fruit extract lyophilized powder was 15,000–58.59 μg/mL, concentration of CM-AgNPs lyophilized powder was 250–0.098 μg/mL, concentration of terbinafine was 0.25–0.00098 μg/mL, and concentration of fluconazole was 64–0.25 μg/mL. Meanwhile, the growth control group (column 10) with only fungal solution added, the control group containing 0.5%DMSO solvent (column 11) and the blank control group (column 12) were set up. Culture at 28°C for 7 d.

#### 2.7.2 MIC determination of *Candida albicans*

*C. albicans* was inoculated on SDA medium and cultured at 35°C for 24 h. The number of spores was determined by cell counting plate, and the concentration was adjusted to 5.9 × 10^4^ CFU/mL by RPMI 1640 medium dilution. MIC was determined according to CLSI-M27 document (Alexander et al., 2017b), the samples were diluted to different concentrations by adding RPMI 1640 culture medium into the 96-well plate (terbinafine was dissolved with DMSO, other drugs were dissolved with deionized water, DMSO content was 1%). In columns 1–9 of the 96-well plates, concentration of *C. monnieri* fruit extract lyophilized powder was 12,500–48.83 μg/mL, concentration of CM-AgNPs lyophilized powder was 25–0.098 μg/mL, concentration of bare AgNPs lyophilized powder was 500–1.95 μg/mL, concentration of fluconazole was 64–0.25 μg/mL, and concentration of terbinafine was 0.5–0.001953 μg/mL. Meanwhile, the growth control group (column 10) with only fungal solution added, the control group containing 1%DMSO solvent (column 11) and the blank control group (column 12) were set up. Culture at 35°C for 24 h.

#### 2.7.3 Result interpretation

Absorption values of 96-well plates at 630 nm were determined using a microplate reader (Infinite E Plex, Tecan Austria Gmbh, Austria), and MIC_90_ of *C. monnieri* fruit extract, bare AgNPs and CM-AgNPs against *T. rubrum*, *T. mentagrophytes* and *C. albicans* were determined by combining visual and microplate detection of percentage inhibition and make a comparison. During the experiment, quality control compounds terbinafine and fluconazole were used as controls, and the MIC fluctuation range was no more than one drug gradient concentration, and the MIC was within the MIC standard range of quality control strains published by CLSI-M38 and CLSI-M27 (Alexander et al., 2017a, 2017b), which was considered reliable experimental data. The experiment was repeated three times and the percentage inhibition was calculated using the following equation:

% inhibition = (A_Growth control_ − A_Sample_) /A_Growth control_ × 100%.

## 3 Results and discussion

### 3.1 Analysis of response surface results

**Figure 1:**
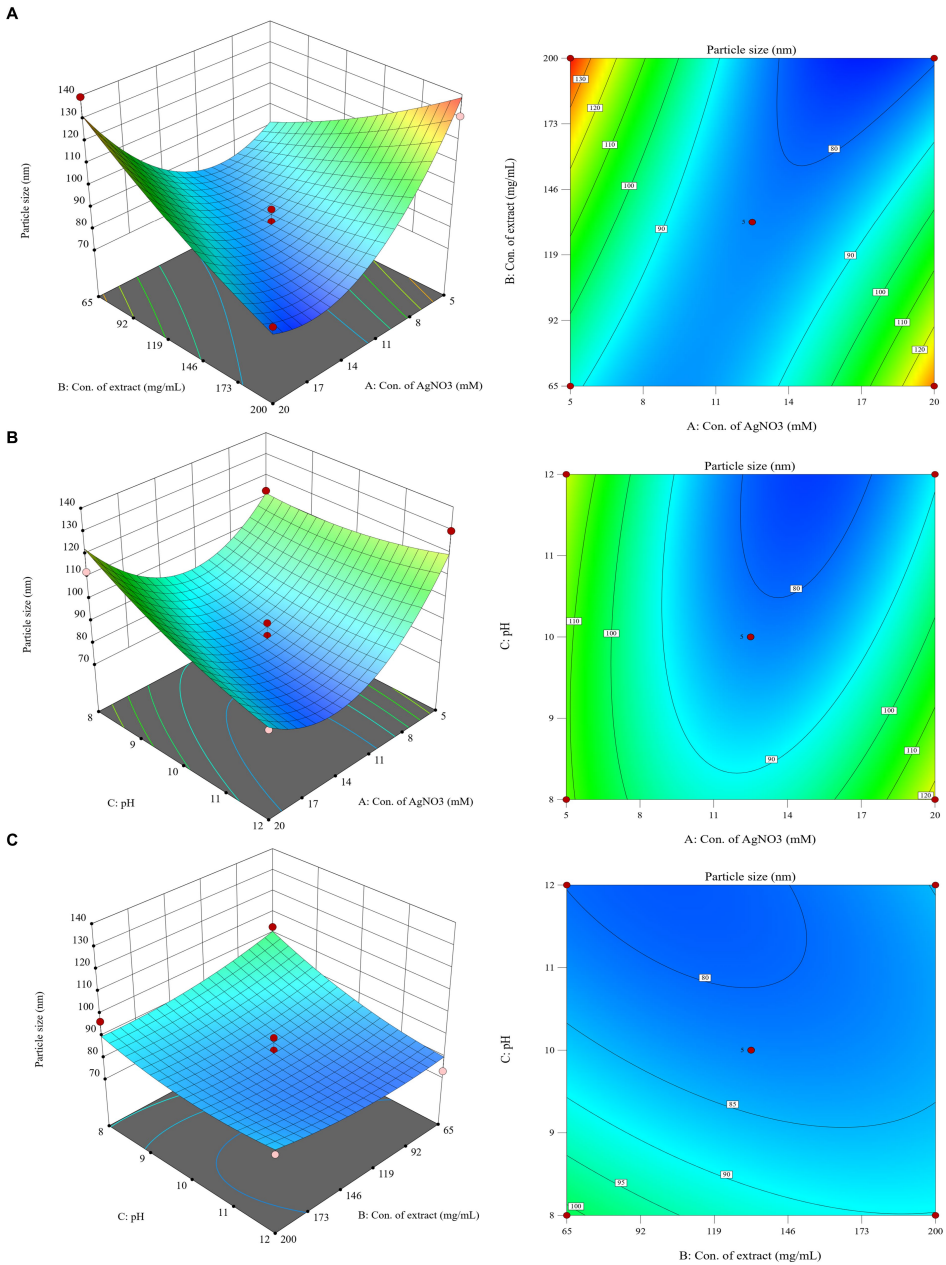
3D response surface plots and 2D contour plots showing the influence of interaction between **(A)**: concentration of AgNO_3_, **(B)**: concentration of *C. monnierii* fruit extract and **(C)**: pH on the particle size of silver nanoparticles.

The synthesized CM-AgNPs were systematically optimized using Box–Behnken design of Design Expert® ver.13.0 software to find the optimal conditions. Concentration of AgNO_3_, Concentration of *C. monnieri* fruit extract and pH were selected as three independent variables at different levels, and the particle size (nm) and PDI of silver nanoparticles were optimized as responses. A total of 17 tests were recommended for the selected design (Table 1). The obtained data were fitted with the quadratic polynomial model, and various statistical parameters were used for fitting analysis. Equations (1) and (2) were polynomial equations generated after modelling as data, indicating that the two response variables analyzed (particle size and PDI) have both interaction and curvature effects. The 3D response diagram (Figure 1) illustrates the good fit of the data in the selected model, where A is concentration of AgNO_3_ (mM), B is concentration of *C. monnieri* fruit extraxt (mg/mL), C is pH value of *C. monnieri* fruit extract.

(1) $$\begin{array}{l} \mathrm{Particle}\;\mathrm{size}=81.91-5.02A-1.40B-6.54C-24.75\mathrm{AB}\\ \;-10.38\mathrm{AC}+4.10BC+25.08A^{2}+3.32B^{2}+3.92C^{2} \end{array}$$

(2) Polydispersityindex=0.3069+0.0423A−0.0427B−0.0088C

For particle size response, ANOVA results show that the model is extremely significant, *p* < 0.01, where AB and A^2^ were important model items. R^2^ = 0.9182, the *F* value of the missing item was 5.71, and the missing item was not significant. For PDI response, ANOVA results showed that the model was significant (*p* < 0.05), where A and B were important model items. R^2^ = 0.5587, the F value of missing item was 1.78, the missing item was not significant, the smaller R^2^ may be due to the fact that PDI results were close to each other in the test range, as they are mostly in the acceptable range below 0.300.

The optimal conditions for CM-AgNPs synthesis were suggested by numerical optimization. The goal of each response variable was to minimize to the smallest possible value. The best conditions were found to be concentration of AgNO_3_ was 18.38 mM, concentration of *C. monnieri* fruit extraxt was 196.77 mg/mL and adjusted to 200 mg/mL, and the pH value of *C. monnieri* fruit extraxt was 11.75. The predicted results showed particle size of 70.1779 nm and PDI of 0.202.

### 3.2 Characterization of optimized silver nanoparticles

#### 3.2.1 UV–vis analysis

**Figure 2:**
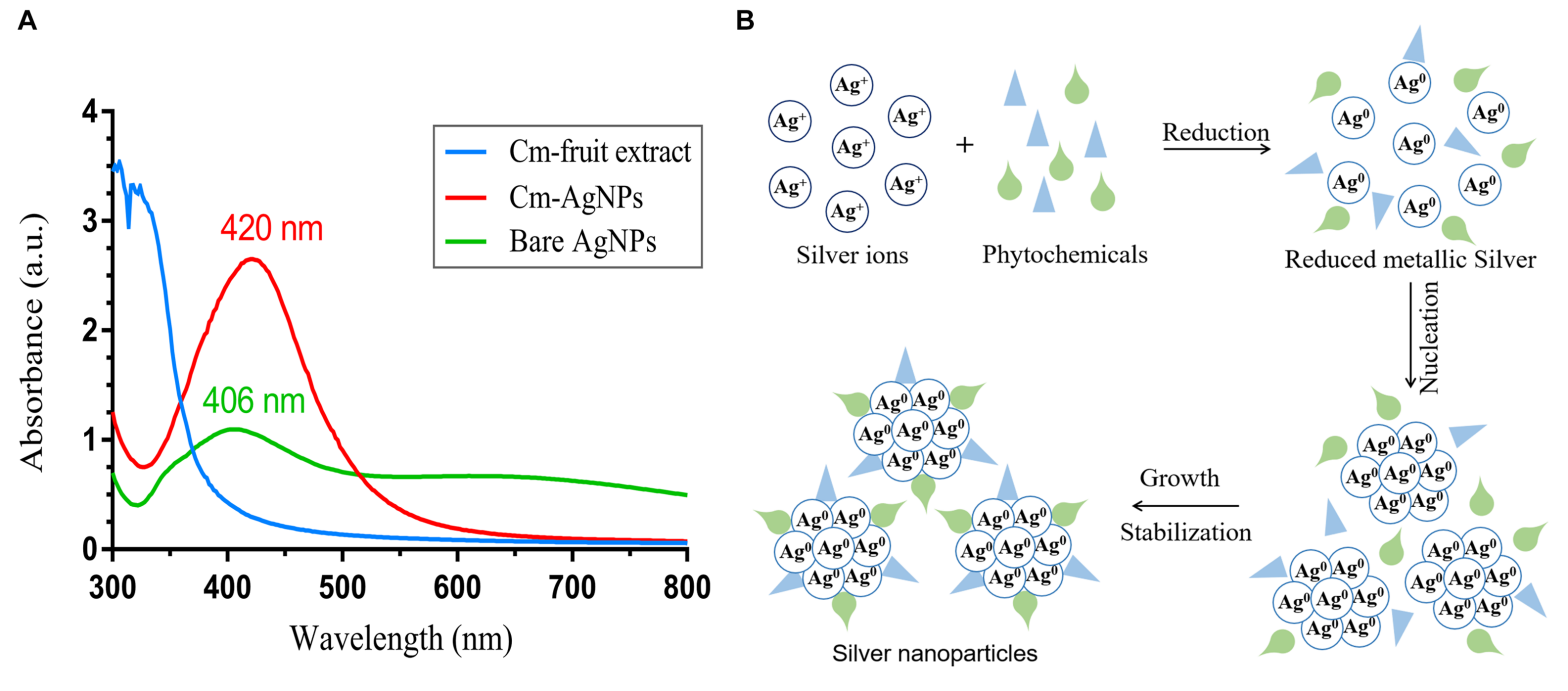
**(A)** UV–Vis spectrum of Cm-fruit extract, CM-AgNPs and bare AgNPs; **(B)** possible mechanism miagram of plant-mediated synthesis of AgNPs.

In this study, AgNPs were synthesized under optimized conditions using *C. monnierii* fruit extract. The plant extract serves as a reducing agent to convert silver ions (Ag^+^) into silver nanoparticles (Ag^0^), termed CM-AgNPs. The synthesis of AgNPS was confirmed through UV–Vis analysis (Figure 2A), with CM-AgNPs exhibiting surface plasmon resonance peak at 420 nm and bare AgNPs exhibiting SPR peak at 406 nm. The position and shape of the displayed SPR peak depend on the particle size and shape of the AgNPs (Rajivgandhi et al., 2019). This experiment synthesized AgNPs by adjusting the pH value, which can accelerate the synthesis and reduce the particle size. Studies have shown that pH value has a certain impact on the synthesis of AgNPs; under alkaline conditions, the hydroxyl groups in plant extracts were more likely to lose H^+^, causing the whole molecule to be negatively charged; these negatively charged phytochemicals not only easier to interact with Ag^+^, but also easier to lose electrons for reduction reaction (Luo et al., 2018). The schematic diagram (Figure 2B) describes the possible mechanism of plant synthesis of AgNPs. Silver ions (Ag^+^) were reduced to form silver atoms (Ag^0^), which slowly aggregated into small silver nanoparticles. During this process, AgNP was restricted by phytochemicals so that the silver could not grow close to each other at the nanoscale and thus form small silver nanoparticles.

#### 3.2.2 FTIR analysis

**Figure 3:**
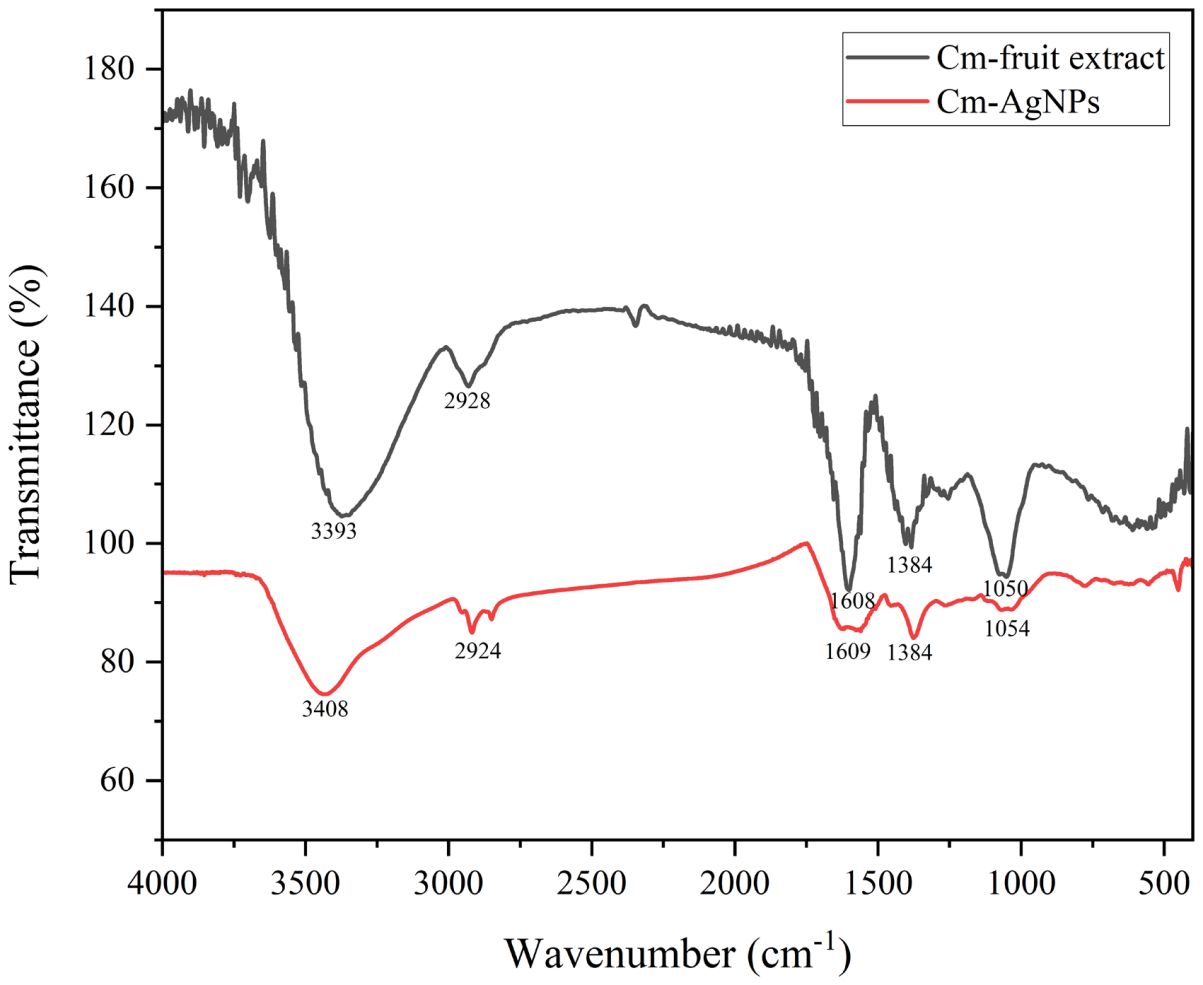
FTIR spectra of Cm-fruit extract and CM-AgNPs.

FTIR analysis was performed to determine the major phytochemicals involved in plant synthesis and sealing of CM-AgNPs. As shown in Figure 3, IR spectra confirmed the binding of silver ions with the extract of *C. monnierii* fruit. CM-AgNPs show the corresponding FTIR signal, O-H (3,408 cm^−1^), C-H (2,924 cm^−1^), benzene ring skeleton (1,609 cm^−1^), -CH_3_ (1,384 cm^−1^) and -C-O (1,054 cm^−1^). These signals matched corresponding peaks in the FTIR spectra of the *C. monnierii* fruit extract. This indicates that many organic functional groups in *C. monnierii* fruit extract actually remained on the surface of CM-AgNPs.

#### 3.2.3 XRD analysis

**Figure 4:**
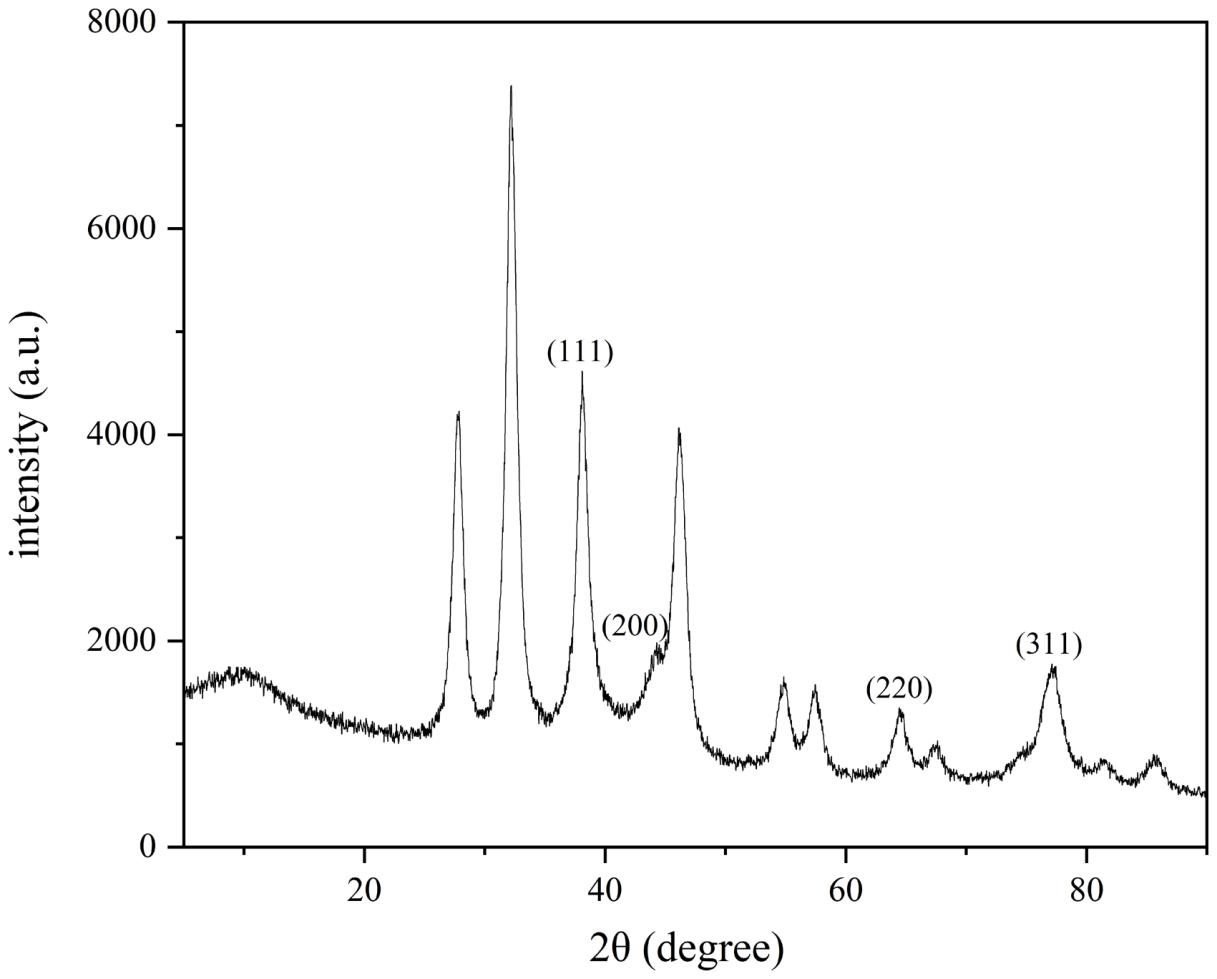
XRD patterns of CM-AgNPs.

The structure of CM-AgNPs was analyzed through XRD measurement (Figure 4). It can be seen that the main diffraction peaks were located at 38.08, 44.24, 64.46, and 77.46 °, pointing to (111), (200), (220), and (311) diffraction planes, respectively. CM-AgNPs showed the diffraction peak characteristics of the metal face-centered cube (JCPDS File No. 4–0783), indicating that the silver nanoparticles formed in this synthesis were essentially crystalline (Zhang et al., 2020; Rajivgandhi et al., 2020b). From the peak intensity ratio of (111) to other diffraction peaks, it can be concluded that the (111) plane was the main orientation in the silver crystal structure of CM-AgNPs.

#### 3.2.4 SEM, EDX, and TEM analysis

**Figure 5:**
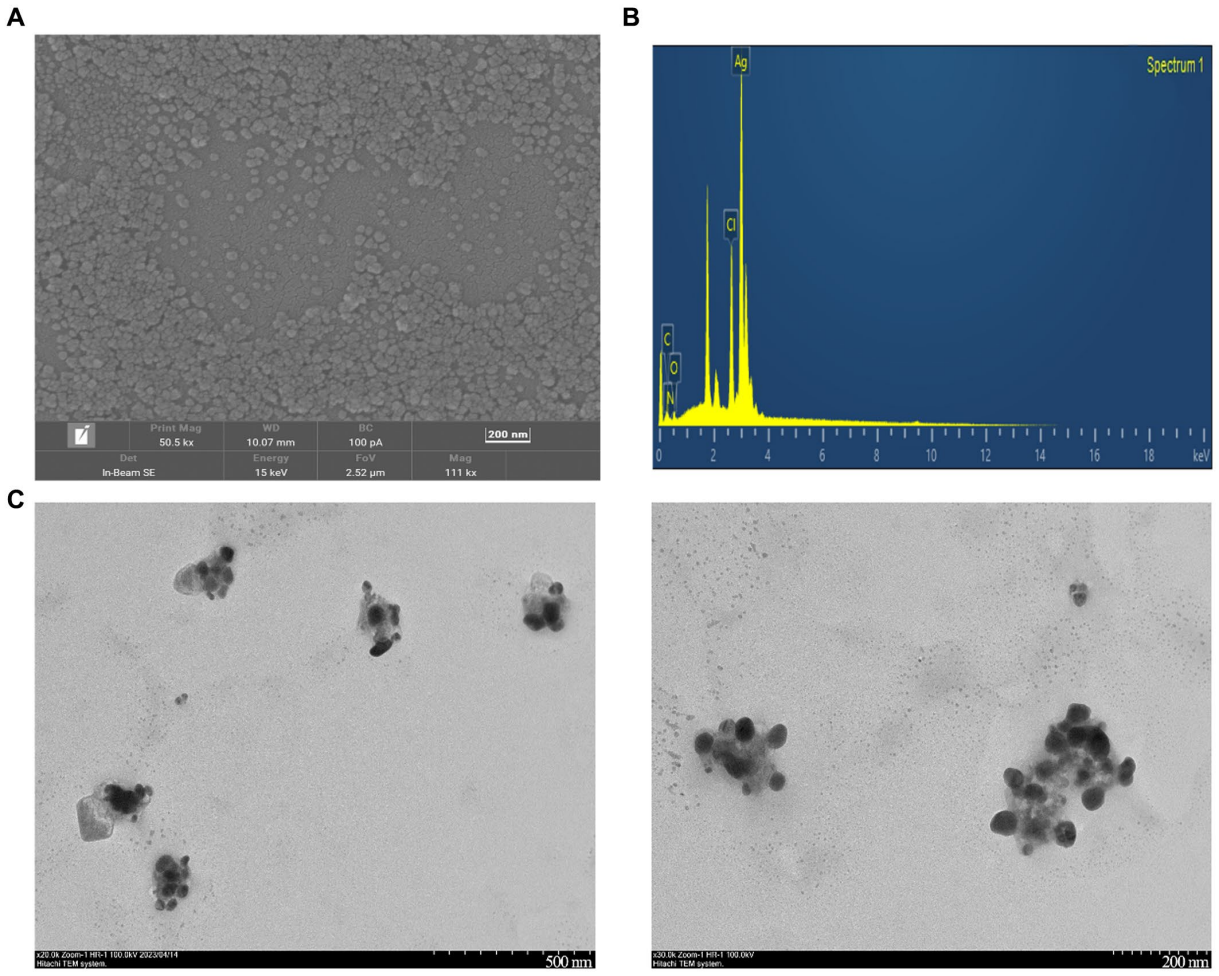
**(A)** SEM images of CM-AgNPs (200 nm); **(B)** EDX spectra of CM-AgNPs; **(C)** TEM images of CM-AgNPs (500, 200 nm).

As shown in Figure 5A, the SEM analysis of CM-AgNPs detected agglomeration, which may be caused by drying the AgNPs solution during detection (Rajivgandhi et al., 2020a). The EDX results indicate that the sample contains silver, which confirms the formation of elemental silver (Figure 5B). The shape and size of the optimized CM-AgNPs can be directly observed by TEM (Figure 5C). It can be seen that the diameter of the synthesized AgNPs was about 44.6 nm and the distribution was uniform. The active ingredients in the extract were attached to the surface of the AgNPs to prevent the aggregation of particles, and the synthesized AgNP was spherical.

#### 3.2.5 DLS and stability analysis

**Figure 6:**
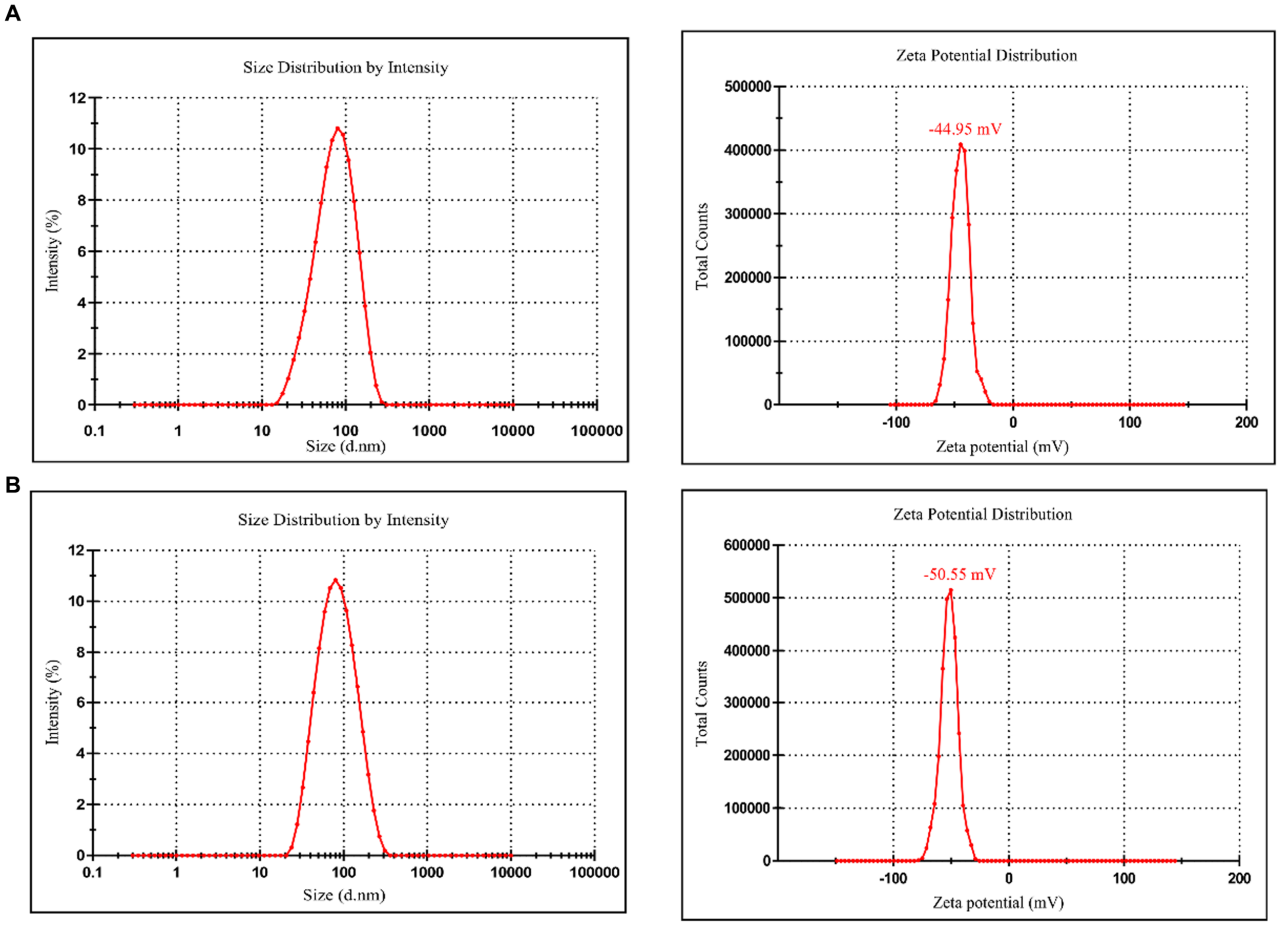
**(A)** Particle size and zeta potential distribution of CM-AgNPs; **(B)** Particle size and zeta potential distribution of bare AgNPs.

Dynamic light scattering (Figure 6A) shows that the optimized average particle size of CM-AgNPs was 56.31 nm, PDI was 0.2375, and zeta potential was −44.95 mV (mean value of three measurements). DLS (Figure 6B) shows that the average particle size of chemically synthesized bare AgNPs was 91.57 nm, PDI was 0.4340, and zeta potential was −50.55 mV (mean value of three measurements). AgNPs were negatively charged on the surface and dispersed in the medium. Electrostatic repulsion between negatively charged nanoparticles may prevent AgNPs from aggregating, which may be responsible for AgNPs stability. This property involved the surface interaction of AgNPs and their effect on cells. DLS used fluid mechanics to detect particle size, so there are some differences between the particle size results and TEM particle size observation results.

After four weeks of synthesis of optimized CM-AgNPs, the detected average particle size was 51.00 nm and PDI was 0.2634. Compared to four weeks ago, the changes were relatively small, indicating good stability of CM-AgNPs.

### 3.3 Analysis of antifungal activity

**Figure 7:**
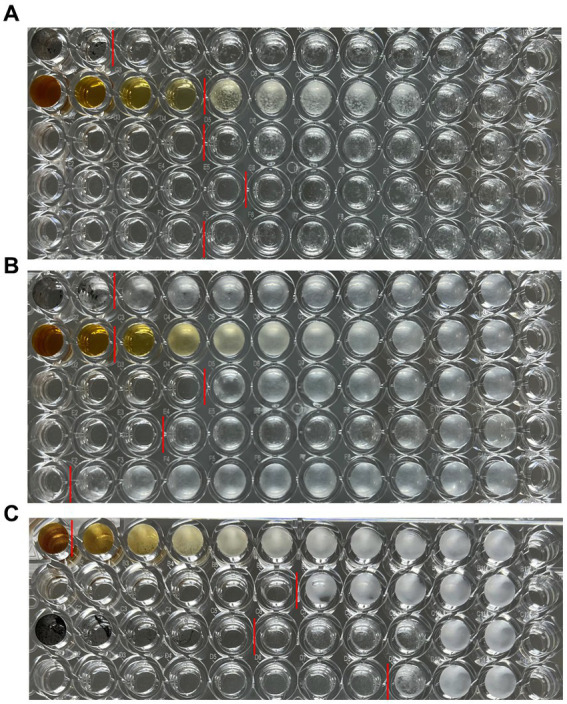
Results of antifungal 96-well plates, **(A)** *T. rubrum*, **(B)** *T. mentagrophytes*, **(C)** *C. albicans*; **(A)** and **(B)** from top to bottom are bare AgNPs freeze-dried powder, *C. monnieri* fruit extract freeze-dried powder, CM-AgNPs freeze-dried powder, terbinafine, and fluconazole; **(C)** From top to bottom, there are *C. monnieri* fruit extract freeze-dried powder, CM-AgNPs freeze-dried powder, bare AgNPs freeze-dried powder, and fluconazole.

After culturing the fungi for 7 days or 24 h, the 96-well plates were removed from the constant temperature incubator and photographed (Figure 7). After the lyophilized powder of *C. monnieri* fruit extract and bare AgNPs were added to the 96-well plate, the dark color affected the detection results of the microplate reader, and the calculated percentage inhibition was not accurate, so two people were used to visually observe the results (Table 2). The minimum inhibitory concentration of CM-AgNPs, terbinafine, and fluconazole with the percentage inhibition greater than 90% calculated by scanning the OD value at 630 nm using microplate reader was MIC_90_ (Table 2). Based on all the results, it can be concluded that the order of efficacy for *T. rubrum* and *T. mentagrophytes* was as follows: terbinafine > CM-AgNPs > fluconazole > bare AgNPs > lyophilized powder of *C. monnieri* fruit extract. Terbinafine has good efficacy against *T. rubrum* and *T. mentagrophytes*, but there are patients with dermatophyte infection who have no clinical response to terbinafine treatment (Bortoluzzi et al., 2023). Therefore, the research and development of alternative therapeutic drugs is significant. For *C. albicans*, the order of efficacy was fluconazole > CM-AgNPs > bare AgNPs > lyophilized powder of *C. monnieri* fruit extract. CM-AgNPs has similar efficacy to fluconazole and is expected to be a potential treatment for *C. albicans*. The MIC of AgNPs synthesized by *Scabiosa atropurpurea* fruit extract against *T. rubrum* and *C. albicans* were 7.81 and 3.9 μg/mL, respectively (Essghaier et al., 2022). Compared with them, the MIC of CM-AgNPs were smaller, which may be due to the better inhibitory effect of *C. monnieri* fruit on ringworm fungi, enhancing the antifungal activity of CM-AgNPs. Studies have shown that AgNPs inhibit fungal growth by affecting fungal morphology, causing membrane infiltration, and producing reactive oxygen species (ROS) to disturb osmotic balance, making cells unstable (Elbahnasawy et al., 2021). AgNPs can also release high affinity silver ions (Ag^+^), inactivating the thiol groups in fungal cell wall, forming insoluble compounds,and then damaging enzymes and lipids bound to the membrane, eventually leading to cell lysis (Astuti et al., 2019).

**Table 2:** MIC_90_ results of various drugs against *Trichophyton rubrum* (7 d), *Trichophyton Mentagrophytes* (7 d), and *Candida albicans* (24 h).

Medicaments	MIC_90_ (μg/mL)		
	*Trichophyton rubrum*	*Trichophyton Mentagrophytes*	*Candida albicans*
Bare AgNPs	125	125	31.25
*C. monnieri* fruit extract	1875	7,500	12,500
CM-AgNPs	3.125	3.125	0.78125
Terbinafine	0.0156	0.0625	–
Fluconazole	8	64	0.5

## 4 Conclusion

In this paper, AgNPs were synthesized from *C. monnieri* fruit, a common Chinese medicine for the treatment of tinea disease. The synthesis method is simple, with small particle size, good stability and low MIC, demonstrating strong medicinal potential for anti dermatophytes. XRD results indicated that CM-AgNPs were face centered cubic (FCC) crystals. TEM showed that the generated nanoparticles were spherical, and the extract of *C. monnieri* fruit was coated on AgNPs, stabilizing the AgNPs, and reducing their aggregation. It was known through FTIR and EDX detection that chemical components such as flavonoids and coumarins in the extract of *C. monnieri* fruit may be involved in the synthesis of CM-AgNPs. For the tested fungi, the anti fungal efficacy of AgNPs synthesized through green synthesis of *C. monnieri* fruit extract was superior to that of chemically synthesized AgNPs, indicating that the extract of *C. monnieri* fruit actually enhanced the antifungal effect of AgNPs. For *C. albicans*, the MIC_90_ of CM-AgNPs was similar to fluconazole. For *T. rubrum* and *T. mentagrophytes*, the antifungal activity of CM-AgNPs was better than that of fluconazole, although weaker than terbinafine. In view of the increased resistance to antifungal agents in clinical candida and dermatophyte infections and the emergence of multiple drug-resistant strains with difficult treatment, the results are of great significance for the development of new topical antifungal drugs, and can be applied in the biomedical field. Cm-AgNPs can be further developed into various topical formulations for the treatment of patients with superficial fungal infections. As a potential alternative treatment strategy, it may help patients cure fungal infections.

## Data availability statement

The original contributions presented in the study are included in the article/supplementary material, further inquiries can be directed to the corresponding authors.

## Author contributions

M-QY: Investigation, Methodology, Writing – original draft. W-WY: Software, Writing – original draft. M-XZ: Formal analysis, Writing – review & editing. H-LH: Resources, Writing – review & editing. A-WH: Conceptualization, Investigation, Validation, Writing – review & editing. BQ: Conceptualization, Writing – review & editing.
