# Corrigendum: Biosynthesis, characterization, and antifungal activity of plant-mediated silver nanoparticles using *Cnidium monnieri* fruit extract

**DOI:** 10.3389/fmicb.2024.1351990

**Published:** 2024-01-19

**Authors:** Mingqi Ye, Wenwen Yang, Minxin Zhang, Huili Huang, Aiwen Huang, Bin Qiu

**Affiliations:** ^1^Fujian University of Traditional Chinese Medicine Fuzong Teaching Hospital (900TH Hospital), Fuzhou, China; ^2^Department of Clinical Pharmacy, 900TH Hospital of Joint Logistics Support Force of PLA, Fuzhou, China; ^3^College of Chemistry, Fuzhou University, Fuzhou, China

**Keywords:** AgNPs, *Cnidium monnieri*, green synthesis, characterization, antifungal

In the published article, there was an error in affiliation 1. Instead of “College of Pharmacy, Fujian University of Traditional Chinese Medicine, Fuzhou, China,” it should be “Fujian University of Traditional Chinese Medicine Fuzong Teaching Hospital (900TH Hospital), Fuzhou, China.”

The authors apologize for this error and state that this does not change the scientific conclusions of the article in any way. The original article has been updated.

